# Semi-automated evaluation of Ki-67 index in invasive ductal carcinoma of the breast

**DOI:** 10.3892/ol.2013.1654

**Published:** 2013-11-04

**Authors:** CRISTIAN SUCIU, ANCA MURESAN, REMUS CORNEA, OANA SUCIU, ALIS DEMA, MARIUS RAICA

**Affiliations:** 1 Department of Microscopic Morphology, ‘Victor Babes’ University of Medicine and Pharmacy, Timişoara, Timiş 300041, Romania; 2 Department of Pathology, Emergency County Hospital, Timişoara, Timiş 300736, Romania; 3 Department of Rehabilitation, ‘Victor Babes’ University of Medicine and Pharmacy Timişoara, Timişoara, Timiş 300041, Romania

**Keywords:** Ki-67 index, counting method, luminal carcinoma, invasive ductal carcinoma, chemotherapy

## Abstract

A significant factor that affects the value of the Ki-67 proliferation index (IK) is the interpretation and implementation approach. This method is based on visual or automated methods to count tumor nuclei labeled with Ki-67 antigen, and is prone to errors. Detection of Ki-67 is a useful tool in breast cancer and contributes to its molecular classification. The current study proposes a method for the quantification of Ki-67-positive tumor nuclei, which allows for the determination of the exact IK value that is required for tumor stratification based on the proliferation rate. The IK was assessed in 81 successive cases of diagnosed invasive ductal breast carcinoma using a semi-automated method that accurately identifies positive tumor cell nuclei. This method prevents the inclusion of other possible positive cells, including lymphoid, normal epithelia and hyperplastic. In small specimens with increased cell density, where the nucleus/cytoplasm ratio is markedly in favor of the nucleus and the distance between nuclei is small, the method allows precise quantification of the nuclei, even when the limits between nuclei are difficult to identify. In addition, images may be stored in a database, including the assessments, and easily accessed when required. We hypothesize that the semi-automated method for counting nuclei offers the most accurate method of assessing the IK and avoids counting errors that may occur through other methods.

## Introduction

Breast carcinoma is the most frequently occurring malignancy in females, representing 22% of all forms of cancer (1). Patients who develop these types of tumor benefit from surgical therapy in association with systemic or local adjuvant therapy, which increases the long-term survival rate. Administration of systemic adjuvant therapy may lead to a number of side effects with major impacts on patient quality of life; therefore, optimal patient selection is necessary (2). The traditional classification of breast carcinoma is based strictly on evident morphological criteria found on tissue preparations, using hematoxylin and eosin staining (3,4). Based on gene expression in the tumor cells of breast carcinomas, Perou *et al*(5) successfully identified several tumor subtypes with specific properties with regard to epidemiology, natural evolution and response to systemic and local adjuvant therapy. Immunohistochemistry (IHC) was used as a surrogate method for molecular subtyping, based on estrogen receptors (ERs), progesterone receptors (PRs), EGFR, HER2 and cytokeratin-5 expression (6–8). Additional studies have shown the necessity of refining criteria for IHC characterization of these tumor subtypes. Currently, in addition to these markers, Ki-67 expression is taken into consideration, enabling quantification of the tumor proliferation index (6).

A significant feature of malignant tumors is their uncontrolled ability to proliferate. Proliferation may be evaluated in various ways, including assessment of the mitotic score by counting mitosis on stained preparations (a mandatory step in determining the histological grade), incorporation of labeled nucleotides into DNA and flow cytometry of the fraction of cells in S phase (9). The most common method used is IHC, which allows for the identification of antigen Ki-67 at the nuclear level using a highly specific antibody. Results are presented as the Ki-67 proliferation index (IK), which represents the percentage of Ki-67-positive tumor cells (9). As shown by Urruticoechea *et al* in 2005 (10), 17 of the 18 studies that included >200 patients showed a statistically significant association between Ki-67 expression and prognosis, providing compelling evidence for a biological correlation. However, the cutoffs to distinguish ‘Ki-67-high’ from ‘Ki-67-low’ varied between 1 and 28.6%, severely limiting its clinical utility. In addition, the numerous steps of the evaluation introduce variability into the results of these assays. Moreover, according to the St. Gallen International Expert Consensus on the Primary Therapy of Early Breast Cancer (2009)(11), breast carcinomas may be stratified into three groups (high, moderate and low) depending on the IK, guiding to a specific therapeutic approach (hormone and/or chemotherapy). In addition, an IK value of 14% represents a threshold for determining the A and B luminal tumors more clearly (6). However, as shown by Recommendations from the International Ki67 in Breast Cancer Working Group, a significant factor that affects the value of the IK is the interpretation and implementation approach. This method is based on visual or automated methods to count labeled tumor nuclei for Ki-67 antigen, and is prone to errors (9,12).

The current study proposes an original method for the quantification of Ki-67-positive tumor nuclei, enabling the determination of the exact value of the IK, which is required for tumor stratification based on the proliferation rate (9). It is a method that may be used for research and diagnostic purposes, which prevents the counting errors that occur with other methods of quantification (12,13).

## Material and methods

### Morphological assessment

A total of 81 consecutive cases of diagnosed invasive ductal carcinomas (IDCs) were examined. IDCs were obtained from the Department of Pathology, Emergency County Hospital (Timisoara, Romania). Specimens were processed using the standard procedure for breast tumors according to World Health Organization (WHO) recommendations (3). Primary processing of tissues (fixation and paraffin embedding) was performed using standard histological techniques. The ethics committee of Ethics committee of ‘Victor Babes’ University of Medicine and Pharmacy approved the protocol of the study and informed written consent was obtained from all subjects according to the World Medical Association Declaration of Helsinki.

From paraffin blocks, 5-μm-thick sections were stained with hematoxylin and eosin and evaluated by two independent pathologists. Quantified conventional parameters included tumor size, histological type and grade, and lymph node status. Based on the assessed parameters, pTNM grading and clinical stage were determined and Nottingham Prognostic Index (NPI) was calculated. Significant tissue fragments were selected from each case and evaluated by IHC. Examination of the slides, morphologically and immunohistochemically, was performed using a Nikon i80 microscope with an acquisition and image processing system (Nikon Instruments Inc., Tokyo, Japan). Tumor size (T) and primary tumor stage were pathologically assessed by three-dimensional measuring; the largest tumor size was used for assessing T stage of pTNM classification. For tumors with an invasive and *in situ* component, only the invasive component was taken into consideration to calculate tumor size (14,15). The average tumor size was 2.8 cm (min, 1; max, 8; median, 2.1). Lymph node status (N) was determined by the evaluation of ≥10 lymph nodes for each case in accordance with WHO criteria for pTNM classification (3). All cases examined ≥15 lymph nodes and no cases showed metastasis (M_0_). Tumor grading was quantified using the histological grading method, which is the most relevant assessment method for IDCs of no specific type (4,16–18). The histological grading system was based on the Scarff Bloom-Richardson (SBR) score, modified by Elston and Ellis (19), which takes into account the degree of differentiation with formation of tubular structures, nuclear pleomorphism and the number of cells in mitosis (15,16). Of the three parameters for SBR score, nuclear pleomorphism is the most subjective, presenting the highest interobserver differences (20). Tubular differentiation was quantified following the examination of all tumor areas, determining the percentage of glandular tubular structures (structures with well-defined lumen) from the total area of the tumor examined. Nuclear pleomorphism was assessed on the least differentiated area (16). Assessment of pleomorphism involves the size of tumor cells relative to normal cells of breast glandular epithelium, presence of nucleoli and their size and chromatin appearance (fine or coarse granular) (17,21). Mitosis counting was achieved on 10 microscope fields, under a ×40 objective (0.59 mm diameter), predominantly found at the periphery of the tumor (avoiding necrotic areas) (21). NPI is based on three parameters, using the following formula: NPI = [tumor size (cm) × 0.2] + lymph node status (score 1, 2 or 3) + histological grade (1, 2 or 3). Tumor size was used in the pTNM staging to assess the primary tumor. Lymph node staging has three levels of assessment, which are as follows: 1, no positive nodes; 2, ≤3 positive nodes (with metastasis); and 3, ≥4 positive nodes or positive apical ganglion. Histological grade was obtained from the SBR score. Numerical values were calculated according to the NPI and its value identifies three prognostic groups, which are as follows: i) good prognostic group (GPG), <3.4; ii) moderate prognostic group (MPG), 3.4–5.4; and iii) poor prognostic group (PPG), >5.4 (16,22). The clinicopathological characteristics of patients with IDC of the breast are shown in Table III.

### IHC data

For IHC evaluation, the most representative paraffin blocks were selected from each case, including primary tumor and non-tumor glandular structures. From each selected block, four sections (3-μm-thick) were used for IHC assessment of ER, PR, HER2 and Ki-67, and staining was performed by the second day following sectioning. Sections were dewaxed and dehydrated, prior to internal peroxidase inhibition with 3% hydrogen peroxide for 5 min. Subsequently, antigen retrieval was performed for 30 min by microwave heating in target retrieval solution (pH 6; DakoCytomation, Glostrup, Denmark), followed by incubation with specific primary antibodies (30 min) using various working systems. 3,3′-Diaminobenzidine hydrochloride was applied for 10 min for visualization, followed by the staining of nuclei with hematoxylin for 3 min. Details of antibodies and the IHC technique are shown in Table I.

### Quantification of IHC reactions

ER- and PR-positive cells were counted using a semi-automated method and all cases with values of >1% were considered positive. Tumors with ER and PR expression levels of ≤50% were considered to have low levels of receptors (low_ER_ and low_PR_), whereas tumors with ER and PR expression levels of >50% were considered to have high levels of receptor expression (high_ER_ and high_PR_). To assess HER2, the Dako HercepTest scoring system was used. The current scoring system accepted by the American Society of Clinical Oncology and College of American Pathologists uses a threshold of ≥30% tumor cells with an intense, continuous and membrane positive reaction for HER2 (23). The reaction to HER2 was scored as follows: 0 (negative), absent or present in <10% of tumor cells; 1+ (negative), membranous, weak and discontinuous in >10% of tumor cells; 2+ (questionable), membranous, low/moderate and continuous in >10% of tumor cells or membranous, intense and continuous in ≤30% of tumor cells; and 3+ (positive), membranous, intense and continuous in >30% of tumor cells. Cases were stratified as luminal breast carcinoma (ER^+^ and/or PR^+^/ ± HER2^+^) and non-luminal breast carcinoma (represented by all ER- and PR-negative cases) (5,7,8,24).

### Ki-67 assessment

**Figure 1:**
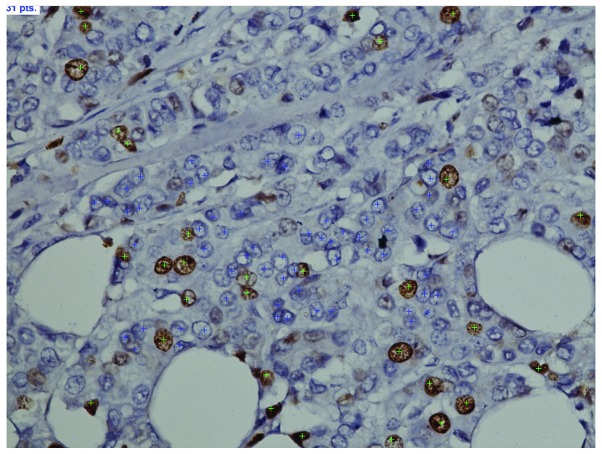
Ki-67 proliferation index calculation was performed using specialized software. Ki-67-positive tumor nuclei are indicated by green markers and negative nuclei are indicated by blue markers (in progress).

Ki-67 assessment was realized by Nikon Eclipse i80 microscope with an image acquisition and processing system, resolution of 2,560×1,920 pixels and color depth of 24 bits (Fig. 1). Each slide was initially examined with a ×10 objective and areas with the highest density of Ki-67-positive nuclei were selected, commonly located close to the periphery of the tumor invasion front. IK was calculated using digital images captured with a ×40 objective, taking into account the current recommendations with regard to the requirement of a minimum number of 1,000 nuclei to be counted for calculating the IK (9).

The tissue fragments labeled with MIB-1 antibody were initially examined with a ×10 objective to identify the areas with the highest density of positive tumor nuclei, commonly located at the periphery of the tumor fragment. Following this, the fragments were examined with a ×40 objective and the necessary number of digital images was captured for each case. IK was calculated from the first digital image to determine the number of tumor nuclei present in the image. From the values obtained, it was clear that a number of digital images were required to be captured for each case to achieve the minimum of 1,000 tumor nuclei to be counted.

Since the number of tumor nuclei counted on a single digital image was an average of 173 (median, 151) and considering the recommendation of counting a minimum of 1,000 nuclei to calculate the IK, an average of seven digital images were captured for each slide (Table II). If following seven captured images the nuclei number was <1,000, additional images were captured to attain the recommended number. A ×40 objective was used for the assessment of the IK and the digital image size captured with the camera had a length of 310.3 μm and a width of 232.72 μm, which corresponds to an area of 0.072213 mm^2^.

**Figure 2:**
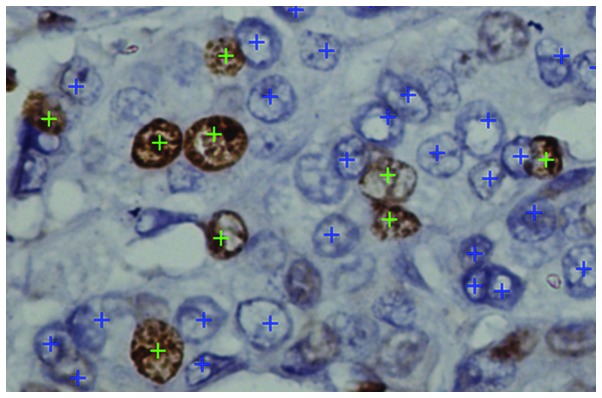
Nuclei counting stage (in progress) using a zoom function, which allowed for the exact identification of positive and negative tumor nuclei.

A special feature in the morphometry software (NIS-Elements D 2.30, Laboratory Imaging s.r.o., Prague), called Counts, allowed for the accurate counting of nuclei in the digital images captured. The computer mouse is positioned on one nucleus belonging to an immunomarked tumor cell (brown) and a marker (green star) is placed on the nucleus (Fig. 1). Automatically, the number of nuclei with markers is counted and appears in a table. Following the completion of counting stained nuclei, the same procedure was used to quantify negative tumor nuclei and cells were automatically recorded into the same table. For high tumor nuclei densities with unclear nuclear limits, a zoom function was used for a more exact selection (Fig. 2).

By identifying the number of stained (immunomarked) and unstained nuclei, the percentage of stained nuclei from the total tumor nuclei counted was calculated, obtaining the IK for each image individually. The end result, implicating the final value of the IK in a particular case, was calculated by taking the arithmetic mean of all the IK values for each image individually. Depending on the value of the IK and considering the criteria proposed by the St. Gallen International Expert Consensus on the Primary Therapy of Early Breast Cancer (2009)(11), luminal carcinoma ER^+^/PR^+^/HER2^−^ may be stratified into exactly three subgroups, which intervene with a specific therapeutic approach. The three subgroups were as follows: IK ≤15%, low_IK_ tumors; IK 16–30%, moderate_IK_ tumors; and IK >30%, high_IK_ tumors.

## Results

### IHC results

Positive immunoreactivity for Ki-67 antigen was localized at the nuclear level with a granular pattern (fine or coarse). The intensity of immunoreactivity varied slightly on this section, without a differentiated quantification of nuclei according to the intensity of reaction. All preparations examined showed normal components (normal glandular lobules) associated with the tumor and were used as internal controls to assess the IHC reaction.

### Quantification of tumor cells

The mean total number of tumor cells counted on a digital image (area, 0.072213 mm^2^) was 173 cells (min, 83; max, 585; median, 151), while the number of Ki-67-positive nuclei had an average value of 45 cells (min,9; max, 134; median,38) (Table II).

### Digital image capture and time required for analysis

In ~50% (n=40) of the cases studied, seven digital images/case was sufficient for the assessment of the number of quantified tumor nuclei to be ≥1,000. In 32% of cases (n=26), it was necessary to evaluate more than seven images/case, with ~50% of these cases (n=12) requiring eight images. For the remaining 18% of the cases (n=15) investigated, 73% (n=11) required five images/case and 27% (n=4) required only two images/case, characterized by high nuclear density (>500 nuclei/image). The time required for tumor nuclei counting, using the proposed method on a digital image containing 173 nuclei, was 2 min and 30 sec and the actual time required to count 1,000 nuclei was 15 min. An additional 5 min (maximum) was necessary for capturing the digital images corresponding to each case as well as ~5 min for calculating the percentage value of the IK. Therefore, the maximum time required for the evaluation of a case was 25 min. The high, moderate and low levels of the IK according to clinicopathological characteristics are shown in Table III.

### IK value analysis

An IK of 68% (n=55) represents ER^+^/PR^+^/HER2^−^ tumors (luminal A), of which specific tumors are likely to be selected for treatment by chemotherapy with a parameter being high_IK_ (Table IV). Following the proposed assessment using a semi-automated method, it was identified that among these tumors only approximately one in five (21.8%) exhibited high_IK_.

As shown in Table IV, a number of ER^+^/PR^+^/HER2^−^ tumors (n=40) were characterized by high_ER_, of which only a small proportion (15%) had high_IK_. This tendency was found to be the case with PR expression. The highest percentage of high_IK_ tumors was characteristic of those with low_ER_ and low_PR_ expression compared with high_ER_ and high_PR_ expression (Table V).

## Discussion

The Ki-67 (MKI67) antigen is a nuclear non-histone protein required for cell proliferation and is encoded by the MKI67 gene, which is located on the long arm of chromosome 10. The antigen was first identified by Gerdes *et al* in 1980 (25) and shortly following this discovery, the anti-Ki-67 antibody was developed. Cellular proliferation involves several defined phases: i) G_0_, resting; ii) G_1_, first gap; iii) S, DNA synthesis; iv) G_2_, second phase of relative inactivity; and v) M, mitotic. Cells may be recycled by entering the G_1_ phase or return to the resting G_0_ phase (10,26). A detailed cell cycle analysis showed that the Ki-67 nuclear antigen is expressed in the G_1_, S, G_2_ and M phases, but is not expressed in non-dividing/quiescent cells that are in G_0_ phase (27). The topographic distribution of Ki-67 also varies during the cell cycle (10,28,29).

Uncontrolled cell proliferation represents the hallmark of malignant tumors and may be assessed by various methods, most commonly by IHC detection of the Ki-67 antigen (30,26). Early breast cancer has a highly variable prognosis and the benefit of existing therapies is often unpredictable. Several morphological parameters, including tumor size, histological grade, vascular invasion and lymph node metastasis, are useful in this regard, but insufficient (8). This has lead to the study of tumor molecular characteristics and currently ER, PR and HER2 are recognized as prognostic and predictive factors (10,12). In addition to these, Ki-67 has been added, as its prognostic role has been recognized by previous studies, particularly when specific subgroups of breast carcinomas have been selected (9,31–33). Moreover, in 2009, Cheang *et al*(34) proposed that a panel of antibodies formed by ER, PR, HER2 and Ki-67 allows for the segregation of the two types of luminal tumors (A and B) taking into account the value of the IK. Thus, identification of the exact value of Ki-67 allows for the differentiation of the two subtypes. The international Ki-67 in Breast Cancer Working Group reported Ki-67 measurement by IHC as the current assay of choice for measuring and monitoring tumor proliferation in standard pathology specimens. However, the group recognized the poor consistency with the precise clinical uses of Ki-67 and the substantial heterogeneity and variable levels of validity in methods of assessment (9,33).

For the clinician, it is important to be aware of exactly what type of systemic adjuvant therapy is administered to patients, as this therapy causes numerous side effects and requires optimal patient selection (2). Therefore, it is necessary to determine: i) which patients are recommended for hormone therapy; ii) which patients are likely to be administered anti-HER2 therapy; and iii) which patients are likely to receive chemotherapy. Administration of endocrine therapy is selected for all cases showing a positive reaction for ER, as the response to therapy is dependent on the level of receptor expression. Anti-HER2 therapy with trastuzumab has been recommended for all HER2-positive tumors (>30% positive tumor cells), according to the American Society of Clinical Oncology and the College of American Pathologists (23). Selection of cases for chemotherapy is the most delicate and difficult step and considers the following patient groups: i) HER2-positive cases, chemotherapy is administered prior to or following the administration of trastuzumab; ii) triple negative tumors, ER^−^, PR^−^ and HER2^−^; and iii) specific ER^+^, PR^+^ and HER2^−^ (luminal) tumors, a subset of tumors receiving hormone and chemotherapy. The precise determination of the subset of patients with luminal carcinomas (ER^+^/PR^+^/HER2^−^) who are suitable for chemotherapy (associated with hormone therapy) is the main issue, and the assessment of IK has shown promising preliminary data with regard to these issues (34,35). According to the St. Gallen International Expert Consensus on the Primary Therapy of Early Breast Cancer, depending on the IK of ER^+^/PR^+^/HER2^−^ tumors, therapeutic management is different. Patients with high_IK_ are a tumor subgroup receiving chemotherapy and hormone therapy, moderate_IK_ patients may benefit from hormone therapy and chemotherapy and low_IK_ patients are likely to benefit only from hormone therapy (11,33). In addition, an IK value of >14% is used to identify luminal B tumors (6). These results show the importance of accurate IK values for all these cases and that low intra- and interobserver variability for IK values are required (33). As shown by Urruticoechea *et al* in 2005 (10), using various antibodies, immunomarking techniques and protocols for assessing score, without a minimum standard with regard to the number of tumor cells to be quantified and optimal thresholds for defining subgroups, are all causes of heterogeneity and obstacles against the use of these methods in clinical practice (9,11,36). Previous studies have shown that limitations for the clinical use of Ki-67 have been due to a lack of standardization concerning the following four groups of parameters: i) preanalytical (type of fixative, time used for fixation and how to preserve the tissue fragments); ii) analytical (use of antigen retrieval, clone of antibody used and the staining of nuclei with hematoxylin); iii) interpretation and implementation of the score (quantification of positive nuclei and/or intensity, assessment of the tumor area and visual/automatic quantification methods); and iv) data analysis (cut point) (9). Tissue fragments to be assessed using the IK were fixed in 10% neutral buffered formalin (3.7% formaldehyde; pH 7.2) for 24 h. It is important to avoid delay in tissue fixation, as the IK value may decrease by 6%. Data from previous studies show that the optimal fixative solution recommended to preserve tissue for IHC evaluation of Ki-67 is neutral buffered formalin and possibly non-buffered formalin. The recommended optimal fixation period is between 6 h and up to 3 days (9), although, there have been previous studies showing that prolonged fixation (154 days) did not significantly reduce Ki-67 immunomarking (37). However, the current study identified that all tumors fixed in unbuffered formalin for >70 days were Ki-67-negative.

Once the tissues are paraffin-embedded, they may be stored at room temperature for a number of years without affecting Ki-67 immunomarking (38). The paraffin blocks used in the current study were selected retrospectively, and the maximum time that passed following the preparation of the blocks and IHC evaluation of Ki-67 was not >2 years. Paraffin sections that are placed on slides and stored at room temperature retain their antigenicity for 3 months (39). Sections stored on glass slides at room temperature for 2 weeks do not change in IK value (9). In the current study, IHC reactions were performed on the second day following the sectioning of the paraffin blocks for Ki-67 and other markers (ER, PR and HER2). With regard to the antibody used for evaluating the IK, the most widely used and recommended is the mouse anti-human Ki-67 monoclonal MIB-1 antibody. Proteases and low pH (<5) must be avoided for antigen retrieval (9,40). The best results are obtained on tissues fixed for a minimum of 6 h and a maximum of 3 days using neutral buffered formalin or non-buffered formalin (pH 5) (41). The working technique of the current study was to fix the specimens for 24 h in neutral buffered formalin at pH 7.2 using clone MIB-1 and antigen retrieval solution at pH 6 (DakoCytomation).

Typically, Ki-67 expression appears at the nuclear level, although, cytoplasmic expression is possible as a result of using MIB-1 antibody, particularly in grade 3, HER2-positive and ER-negative breast cancer with squamous metaplastic changes (42). However, this is not a serious issue for interpretation; for the IK, appreciation of nuclear expression is taken into account, which is rarely masked by cytoplasmic reactions. Scoring systems are based on the percentage of tumor cells stained by the antibody. This requires counting ≥1,000 tumor cells with nuclear staining under a high-powered field (magnification, ×40) with laboratory limitations. Certain pathologists estimate the percentage of nuclei staining, whereas others count several hundred consecutive nuclei in various areas of tumors to determine an overall average index. However, estimating the percentage of cells is poorly reproducible and manual counting is tedious, with high interobserver variability (12,13,43). Therefore, automated readers have been used for scoring large series of samples (44). A significant concern is that automated methods may count non-malignant nuclei, whereas a manual count is likely to exclude this potential error. There are also significant discrepancies of the IK value in these cases when using automated and visual methods, particularly in tumors with heterogeneous Ki-67 expression (45). The proposed semi-automated method excludes errors and technical issues that may occur by visual and automated counting methods.

Advantages of the semi-automated method are as follows: i) accurate identification of positive tumor nuclei, preventing the counting of other possible positive cells, including lymphoid, normal epithelial and hyperplastic (12); ii) precise quantification of nuclei, even when the limits between nuclei are difficult to identify when tumor fragments have an increased cell density where the nucleus/cytoplasm ratio is clearly in favor of the nucleus and the distance between nuclei is small; iii) precise quantification of negative tumor nuclei, preventing the counting of any other non-tumor cells, including increased nuclear density areas; iv) storage of images in a database, including the assessments, with easy access to data when required; and v) it may be used on virtual slides in telepathology. It is likely that the only disadvantage of the semi-automated method is that it is time consuming, however, the trained pathologist is able to make this count relatively rapidly (maximum, 25 min/case). This method requires the use of a digital image acquisition and processing optical system, however, pathology departments and research centers where the evaluation of the IK is performed have these systems, and therefore, this method may be used routinely.

We hypothesize that the semi-automated method for counting nuclei offers the most accurate method of assessing the IK and avoids counting errors that may occur through other automatic or manual counting methods. This method is used to assess the rate of proliferation in IDC (and other non-mammary tumors) in our institutions.

The current study proposes the use of this method for the evaluation of any nuclear marker that requires the quantification of breast carcinomas, particularly ER, PR, AR, p53 and other markers. In addition, the semi-automated method may be used to identify the tumor proliferation rate of all tumor entities (other than breast), which requires the determination of the IK by using the methodology adapted for the specific evaluation of tumors.

## Figures and Tables

**Table I:** Antibodies and working systems used.

Marker	Clone	Source	Dilution	HIER, min (pH)	WS
ER	1D5	DakoCytomation^a^	RTU	MW, 30 (6)	LSAB-HRP
PR	PgR636	DakoCytomation^a^	RTU	MW, 30 (6)	LSAB-HRP
HER2	Polyclonal	DakoCytomation^a^	RTU	MW, 30 (6)	EnVision-HER
Ki-67	Monoclonal, MIB-1	DakoCytomation^a^	RTU	MW, 30 (6)	LSAB-HRP

a DakoCytomation, Glostrup, Denmark.

HIER, heat-induced epitope retrieval; WS, working system; ER, estrogen receptor; PR, progesterone receptor; RTU, ready-to-use; MW, microwave; LSAB-HRP, labeled streptavidin biotin-horseradish peroxidase.

**Table II:** Number of evaluated tumor nuclei and nuclear density.

	Mean	Median	Min/max
Total tumor nuclei, n^a^	173	151	83/585
Ki-67-positive nuclei, n^a^	45	38	9/134
Nuclear density, n/mm^2^	2451	2091	1149/8101

a Number of nuclei evaluated on a single digital image, corresponding to an area of 0.072213 mm^2^, using a ×40 objective.

**Table III:** IK distribution according to clinical and pathological criteria.

Criteria	Total, n	High_IK_,% (n)	Moderate_IK_, % (n)	Low_IK_, % (n)	IK
IDC	81	37.1 (30)	25.8 (21)	37.1 (30)	30.2
Pre-menopause, ≤50 years	30	40.0 (12)	30.0 (9)	30.0 (9)	33.2
Post-menopause, >50 years	51	35.3 (18)	23.5 (12)	41.2 (21)	28.4
Histological grade					
G1	9	0.0 (0)	0.0 (0)	100.0 (9)	12.6
G2	45	20.0 (9)	33.3 (15)	46.7 (21)	24.8
G3	27	77.8 (21)	22.2 (6)	0.0 (0)	44.9
Lymph node metastasis					
Yes	51	47.1 (24)	17.6 (9)	35.3 (18)	31.3
No	30	20.0 (6)	40.0 (12)	40.0 (12)	28.3
IDC					
Luminal	63	19.1 (12)	33.3 (21)	47.6 (30)	21.7
Non-luminal	18	100.0 (18)	0.0 (0)	0.0 (0)	59.6
Stage					
I	12	0.0 (0)	25.0 (3)	75.0 (9)	13.7
II	33	45.4 (15)	27.3 (9)	27.3 (9)	35.3
III	36	41.7 (15)	25.0 (9)	33.3 (12)	31.1
NPI					
GPG	24	12.5 (3)	25.0 (6)	62.5 (15)	19.1
MPG	30	40.0 (12)	30.0 (9)	30.0 (9)	33.2
PPG	27	55.6 (15)	22.2 (6)	22.2 (6)	36.7

IK, Ki-67 proliferation index; IDC, invasive ductal carcinoma; NPI, Nottingham Prognostic Index; GPG, good prognostic group; MPG, moderate prognostic group; PPG, poor prognostic group.

**Table IV:** IK distribution in ER^+^/PR^+^/HER2^−^ luminal tumors depending on low or high levels of ER and PR.

Levels of ER and PR	High_IK_, % (n)	Moderate_IK_, % (n)	Low_IK_, % (n)	Total, n
Cases (ER^+^/PR^+^/HER2^−^)	21.8 (12)	36.4 (20)	41.8 (23)	55
ER				
High	15.0 (6)	42.5 (17)	42.5 (17)	40
Low	40.0 (6)	20.0 (3)	40.0 (6)	15
PR				
High	18.8 (6)	43.7 (14)	37.5 (12)	32
Low	26.1 (6)	26.1 (6)	47.8 (11)	23

IK, Ki-67 proliferation index; ER, estrogen receptor; PR, progesterone receptor.

**Table V:** IK distribution in ER^+^/PR^+^/HER2^−^ luminal tumors depending on combined levels of ER and PR (high and low).

Cases (ER^+^/PR^+^/HER2^−^)	High_IK_, % (n)	Moderate_IK_, % (n)	Low_IK_, % (n)	Total
High_ER_ and high_PR_	13.1 (3)	60.8 (14)	26.1 (6)	23
High_ER_ and low_PR_	17.6 (3)	17.6 (3)	64.8 (11)	17
Low_ER_ and high_PR_	33.3 (3)	0.0 (0)	66.7 (6)	9
Low_ER_ and low_PR_	50.0 (3)	50.0 (3)	0.0 (0)	6

IK, Ki-67 proliferation index; ER, estrogen receptor; PR, progesterone receptor.
